# Differential hippocampal and retrosplenial involvement in egocentric-updating, rotation, and allocentric processing during online spatial encoding: an fMRI study

**DOI:** 10.3389/fnhum.2014.00150

**Published:** 2014-03-20

**Authors:** Alice Gomez, Mélanie Cerles, Stéphane Rousset, Chantal Rémy, Monica Baciu

**Affiliations:** ^1^LPNC, Université Grenoble AlpesGrenoble, France; ^2^CNRS, LPNC UMR 5105Grenoble, France; ^3^ESPE, Centre de Neurosciences Cognitives, UMR 5229, Université Claude Bernard Lyon 1Bron, France; ^4^Joint Service Unit, UMS 3552, ‘IRMaGe’, CNRS/INSERM, Grenoble Institute of Neuroscience, Joseph-Fourier UniversityGrenoble, France; ^5^Team 5 “Functional Neuroimaging and Brain Perfusion” of Grenoble Institute of Neuroscience, INSERM/CEA, Joseph Fourier UniversityGrenoble, France

**Keywords:** hippocampus, retrosplenial, allocentric, egocentric, navigation, rotation

## Abstract

The way new spatial information is encoded seems to be crucial in disentangling the role of decisive regions within the spatial memory network (i.e., hippocampus, parahippocampal, parietal, retrosplenial,…). Several data sources converge to suggest that the hippocampus is not always involved or indeed necessary for allocentric processing. Hippocampal involvement in spatial coding could reflect the integration of new information generated by “online” self-related changes. In this fMRI study, the participants started by encoding several object locations in a virtual reality environment and then performed a pointing task. Allocentric encoding was maximized by using a survey perspective and an object-to-object pointing task. Two egocentric encoding conditions were used, involving self-related changes processed under a first-person perspective and implicating a self-to-object pointing task. The *Egocentric-updating condition* involved navigation whereas the *Egocentric with rotation only condition* involved orientation changes only. Conjunction analysis of spatial encoding conditions revealed a wide activation of the occipito-parieto-frontal network and several medio-temporal structures. Interestingly, only the cuneal areas were significantly more recruited by the allocentric encoding in comparison to other spatial conditions. Moreover, the enhancement of hippocampal activation was found during *Egocentric-updating encoding* whereas the retrosplenial activation was observed during the *Egocentric with rotation only* condition. Hence, in some circumstances, hippocampal and retrosplenial structures—known for being involved in allocentric environmental coding—demonstrate preferential involvement in the egocentric coding of space. These results indicate that the raw differentiation between allocentric versus egocentric representation seems to no longer be sufficient in understanding the complexity of the mechanisms involved during spatial encoding.

## Introduction

In order to interact with space, humans need to integrate spatial information according to new objects encountered and/or new motion information (e.g., object- or self-motion, Wolbers and Hegarty, 2010). Encoding and storing spatial information is supposed to rely on the activity of one of these two types of spatial representation (Burgess, 2006): (a) egocentric, centered on the subject, coding for self-to-object relations; (b) allocentric, centered on the environment, coding for object-to-object relations. Various evidence sources, ranging from single neuron recordings in animals (O'Keefe and Dostrovsky, 1971; Andersen et al., 1985) to fMRI and neuropsychological observations (Bisiach and Luzzatti, 1978; Aguirre and D'Esposito, 1999; Holdstock et al., 2000; Mellet et al., 2000; Spiers et al., 2001; Chokron, 2003; Burgess et al., 2006; Fields and Shelton, 2006; Maguire et al., 2006; Shrager et al., 2008), suggest that the two types of spatial representations rely on the occipito-temporo-parietal cortices. Among these regions, the posterior parietal cortex sustains egocentric processing (Chokron, 2003) while the medio-temporal regions are required for the allocentric processing (O'Keefe and Nadel, 1978; Holdstock et al., 2000; Milner and Goodale, 2008; Finke et al., 2011).

The connection proposed between the allocentric processing and the hippocampus is partly due to the strong relationship between the “Place cells” firing and landmark sensory aspects observed in animal studies (e.g., O'Keefe and Dostrovsky, 1971; O'Keefe and Nadel, 1978; O'Keefe and Burgess, 1996). fMRI studies in humans during route navigation congruently revealed that the activation of hippocampal and parahippocampal regions correlated with relevant landmark information (Janzen and Van Turennout, 2004; Janzen and Weststeijn, 2007; Janzen et al., 2007). However, several data sources suggest that the hippocampus is not always involved or necessary for allocentric processing (Aguirre et al., 1996; Bohbot et al., 1998; Galati et al., 2000; Committeri et al., 2004; Bastin et al., 2013). For instance, Bohbot et al. (1998) reported, that the performance of patients suffering from unilateral hippocampal lesions in an adapted version of the Morris water maze task (assessing landmark-based allocentric representation) was similar to that of control subjects. Moreover, as revealed by fMRI, some variants of allocentric processing seem to be supported by a large network of regions including parietal, retrosplenial, and parahippocampal cortices (e.g., Aguirre et al., 1996; Vallar et al., 1999; Galati et al., 2000; Committeri et al., 2004; Zhang and Ekstrom, 2013; for review Galati et al., 2010).

The observable discrepancy between these results and the exclusive allocentric role proposed for the medio-temporal lobe (O'Keefe and Dostrovsky, 1971; O'Keefe and Nadel, 1978) could be due to the way spatial information is encoded. Specifically, beyond the allocentric vs. egocentric distinction relative to the type of spatial relation involved (object-to-object or self-to-object, respectively), the presence or the absence of self-related changes could be a decisive factor in the hippocampal involvement. Using a short-term spatial task, Gomez et al. (2014, 2012) disentangled different sources of information required during encoding. The authors showed that patients suffering from bilateral hippocampal atrophy were impaired for reproducing a trajectory learned on the basis of self-motion information. However, they performed similarly to controls when the trajectory was learned without any self-related changes (i.e., observing the experimenter producing the trajectory). This result is in-line with the hypothesis that hippocampal activity may be observed when self-related changes are required to build spatial representations.

Functional MRI evidence provides indirect support to this last hypothesis. Studies manipulating the type of visual perspective during spatial encoding itself showed that hippocampal and parahippocampal structures were more activated during route navigation than during survey navigation (Shelton and Gabrieli, 2002; Shelton and Pippitt, 2007). Hence, despite previous behavioral reports of a strong connection between survey perspective and allocentric representation formation (Thorndyke and Hayes-Roth, 1982; Allen, 1999), such processing seems less tightly related to hippocampal activity than during route navigation. The enhanced activation of hippocampal and parahippocampal structures for route navigation may reflect the integration of new information generated by self-related changes during the egocentric spatial processing (Shelton and Pippitt, 2007).

Beyond the type of visual perspective during spatial encoding, the participants' spatial processing during each encoding condition should be further controlled to insure that the differential hippocampal activity is related to new self-related changes. Actually, the foreseen retrievals tasks can modulate the strategies (allocentric vs. egocentric) used by participants during encoding itself (Shelton and Gabrieli, 2004). From a methodological point of view, this control can be achieved by warning participants that they will have to perform an object-to-object relation retrieval of the spatial information before they enter the survey perspective encoding. On the contrary, participants can be instructed to encode spatial information to subsequently perform a self-to-object relation retrieval task which will insure that the two tasks will in fact differ on the integration (or not) of self-related changes.

If the presence/absence of self-related change is indeed crucial, the information about how the self-related changes are generated could be relevant in providing a thorough understanding of the mechanisms responsible for the different involvements in the spatial memory network. Specifically, given areas could be involved when a change in head direction modifies orientation cues without updating the observer's location. On the reverse, different ones could be involved when both a change in orientation cues and an update in the observer's position occur.

Regarding the head orientation, studies on single cell recordings in animals revealed that several cells fired selectively when the animal oriented its head in a given direction. Cells' localization was first described in post-subiculum (Ranck, 1984), limbic system (Taube, 1998; Robertson et al., 1999) including the retrosplenial cortex (Cho and Sharp, 2001), and hippocampus (Leutgeb et al., 2000) of animals. In humans, heading disorientation was observed after lesion of the retrosplenial cortex (Aguirre and D'Esposito, 1999). More recently, using single-neuron recordings, Jacobs et al. (2010) showed that human entorhinal cortex could contain path cells encoding in a clockwise or counterclockwise route direction during navigation in circular environments. Functional MRI studies have revealed that retrosplenial activity was modulated by previously learnt variations of the head direction (Baumann and Mattingley, 2010).

Farrell and Robertson (1998) defined as the egocentric-updating processing, changes related to both head orientation (i.e., head direction changes) and observer's spatial position. Several animal studies and models of spatial neuronal networks have involved hippocampal “Place cells” during such spatial changes. In fact, based on neuronal recordings in animals, these models indicate that place cell firing is also related to path integration (Mittelstaedt and Mittelstaedt, 1982), which refers to the ability to return directly to a starting point from any location relying mainly on idiothetic cues (i.e., self-motion cues). Path integration can be considered as a subtype of the general process of egocentric-updating where objects are updated relative to the individual (Burgess, 2008). These models emphasized the need to combine landmark sensory aspects and idiothetic cues to build a place code (e.g., Redish and Touretzky, 1997; McNaughton et al., 2006).

These various approaches suggest that the retrosplenial cortex may be crucial in processing heading changes within egocentric referencing, while the hippocampus may be related to egocentric-updating. No fMRI evidence in humans has clearly disentangled the underlying network subtending these two types of processing.

In the present fMRI study, we aim to characterize the neural substrate of spatial memory encoding. Beyond an allocentric (object-to-object) vs. egocentric (self-to-object) distinction, we further disentangled the egocentric information by varying the types of self-related changes during encoding (egocentric-updating vs. rotation only). As such, we defined three spatial encoding conditions: Allocentric, Egocentric-updating, and Egocentric with rotation only.

One hypothesis is that the cerebral organization evolution of cognitive functions from animals to humans could lead to a different neural specialization of spatial memory encoding in specific areas such as the hippocampal and retrosplenial areas. Alternatively, given the neuropsychological evidence, we expect that spatial cognitive functions share similar structures in animals and humans. Therefore, we can expect that the egocentric-updating condition reveals additional activity in the hippocampal structure compared to the allocentric condition. This supports the idea that the human hippocampal area could also code for self-related changes. Additionally, compared to egocentric-updating, the egocentric with rotation only condition should involve retrosplenial activation, supporting the idea that the human retrosplenial cortex codes specifically for orientation changes.

## Materials and methods

The experiment consisted of two phases: a training phase performed outside the MR magnet and a testing phase performed during the MR examination. The training phase had three objectives: (1) familiarization with the virtual presentation *via* free navigation in the environment, (2) instructions on how to perform each task, (3) task execution with performance feedback. During the testing phase, three spatial encoding conditions and a control condition were performed during a block-design paradigm.

### Participants

Eighteen adults (age range 17–30, average age 23.5 SD 2.5, 13 males) took part in the experiment. All participants were right-handed according to the Edinburgh Handedness Inventory (Oldfield, 1971). They had normal or corrected-to-normal vision and no history of neurological or psychiatric disorders. They gave their informed written consent for the experiment and the study was approved by the local ethic committee (CPP n°08-CHUG-10, 20/05/2008).

### Spatial environment, spatial layouts, and encoding films

A Virtual Reality Markup Language (VRML) was used to create the spatial environment. This virtual environment was a 9 × 9 × 3 m room with stone walls. Tile flooring for the southern half and wood flooring for the northern half provided easy orientation in the environment. Each environment contained 6 objects. Twenty different spatial layouts were created by randomly changing the 6 objects' positions. Spatial layouts presenting familiar configurations such as lined-up objects were removed. An in-house VRML-Prime software was created (http://webu2.upmf-grenoble.fr/LPNC/membre_eric_guinet) with the following characteristics: (1) joystick-navigation in the environment, (2) online joystick data recording, and (3) joystick-data-based feedback. To control what the participants see, the VRML-Prime was used to create films of the pre-determined layouts. VRML-Prime made it possible to switch independently between: (1) the visual perspective of the environment (aerial or ground-level) and (2) the type of camera movement in the environment (i.e., rotation only, route navigation, or sequential map presentation).

**Figure 1:**
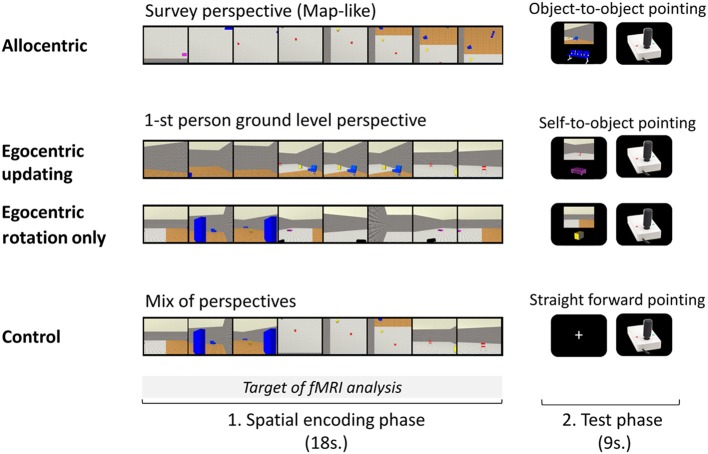
**A trial of each experimental condition performed during the experimental phase inside the MR imager is presented**. Each of the four conditions, A, Allocentric; EU, Egocentric-updating; ERO, Egocentric with Rotation Only; and C, Control; was divided into an encoding phase and a test phase. In the encoding phase, participants saw a presentation of the environment's layout: (A) from a survey perspective, (EU) from a 1-st person ground level perspective with navigation, (ERO) from a 1-st person ground level perspective with a rotation only (C) with a mix of perspectives. In the test phase, participants had to point in the direction of an object using a joystick: (A) from the location of another object, (EU and ERO) from their body position; in condition C, they pointed straightforward.

Three visual spatial encoding conditions were created (See Figure 1): Allocentric (A), Egocentric-updating (EU), and Egocentric with Rotation Only (ERO). For the A films (See Video 1, Gomez, 2014a, http://figshare.com/articles/Allocentric_video_example/902846), a survey perspective (i.e., a bird's eye perspective, looking straight down, with 15% of the environment visible at any moment) was adopted; the camera scanned the map of the environment with an unchanging orientation. This viewpoint was selected to allow the average amount of environment visible at any given point to be equivalent to the ground-level condition. In addition, pilot studies have indicated that this viewpoint induced participants to spontaneously perceive this as “the map” condition. For the EU films (See Video 2, Gomez, 2014b, http://figshare.com/articles/Egocentric_updating_video_example/902847), a ground-level 1st person perspective was adopted; the camera movement was used to simulate the view of an observer walking through the environment. For ERO films (See Video 3, Gomez, 2014c, http://figshare.com/articles/Egocentric_with_Head_rotation_video_example/902848), a ground-level 1st person-perspective (i.e., looking straight from the perspective of a 1 m 80 tall observer) was adopted; the camera movement offered a rotation of 180°, from a fixed location (i.e., one side of the room). Each one of the 20 spatial layouts, resulted in three encoding films that lasted 17700 ms. A fourth control category of films was created using a mix of the ERO, EU, and A films: about 6 s of each of the three films were selected and pooled together in a random order, thus resulting in a 17700 ms control film. The camera movement simulated a path of about 20 m with one or two direction changes. Given that self-motion perception in virtual environments is most accurate when displacement velocity resembles natural locomotion, we adopted a speed of a moderately paced walk (approximately 1.5 m/s). The path and speed for the aerial and ground films were the same. The layout configuration presented in each movie was always different and contained an average of 5 objects (range 4–6, as all 6 objects from an environment were not visible in each movie). The encoding conditions for a given spatial layout were randomly assigned for each participant.

### Experimental conditions: the spatial tasks

Each spatial task trial was composed of an encoding and an immediate test phase (See Figure 1).

In the encoding phase, a film (one of the four types A, EU, ERO, and control, C) was presented to the participants and they were asked to encode and update with the sequential presentation the objects' positions of the objects in the environment. The A film presented a survey perspective of the environment, the ERO movie presented a 1st person rotation from an unchanged location, the EU movie presented a 1st person perspective navigation in the environment and the C movie was a mixture of the three spatial movies which made the control for purely visual stimulations possible. The film presentation was preceded by a 300 ms fixation point included in the “encoding phase.” Participants' spatial knowledge was then tested to control for attention and spatial processing during the encoding phase.

In the test phase, participants were instructed to locate the direction of an object, and to point in its direction with the joystick when prompted. To enhance each type of spatial processing during the encoding phase, participants were told beforehand which test will follow. In the A condition, the A referencing was maximized by asking participants to point in the direction of an object relative to another object in the fixed referencing of the environment (i.e., object-to-object pointing relative to the fixed orientation of the map). Participants were instructed to imagine that they were sketching the direction on the map of the environment. The egocentric referencing was maximized by asking participants to point in the direction of an object relative to their position (i.e., self-to-object pointing), in the EU and ERO conditions.

Each test phase of a trial lasted 9 s: first a question screen was presented during 6 s, and then a joystick picture was presented during 3 s. The joystick picture (320 × 256 pixels) presented on a black screen, served to prompt the participants' response and to collect behavioral performance. The question screen presented (1) the origin of the spatial referencing and (2) the object-to-be-pointed-to on a black background. The origin of the spatial referencing was presented by a 1st person point of view of the environment in the egocentric referencing. The participant could thus simulate its position in the environment from the perspective seen (the point of view was extracted from the previous film). In the A referencing, the point of view was replaced by the presentation of a second object with contextual information from the background. Both the origin and the object-to-be pointed-to were selected from the first and second half of the film respectively, and separated by a minimum of 9 s in the film presentation. They were presented centered on a black background, with the origin at the top (896 × 670 pixels picture), and the object-to-be-pointed-to at the bottom (512 × 410 pixels picture). For condition C, the test was aimed to control for motor preparation, and participants were asked to point straight in front of them (i.e., 0° angle).

### Experimental procedure

As mentioned previously, the experimental procedure was composed of two phases: a training phase outside the magnet and an experimental phase during fMRI.

#### Training phase outside the MR imager

Participants were first asked to navigate freely within an environment designed for this phase using the VRML-prime software. The purpose of this step of the training procedure was to familiarize participants with the use of the joystick, the virtual desktop presentation and the 6 objects.

Then, participants were trained in the experimental conditions previously described. An example of each spatial task was presented (A, EU, ERO, and C), and participants were instructed how to perform each of the four tasks. During the training phase, the test phase of a trial was completed by a visual feedback on the pointing response (from online data recording). In the feedback of the A training condition, the feedback screen displayed the entire map of the environment, with (1) a green arrow (i.e., correct answer) pointing from the origin object toward the expected target object and (2) a blue arrow (i.e., given answer) pointing from the origin object toward the given direction. The error-angle was thus shown as the angle between each of these arrows. The smaller the angle, the more precise the response. This feedback procedure proved successful since all participants were subsequently able to perform the tasks with relatively low pointing errors. In the feedback of the EU and ERO training conditions, a 1st-person perspective video was presented, showing a rotation from a fixed location. The camera was placed on the original location facing the given answer direction. It then rotated until it was in line with the direction of the expected answer. It thus provided an angular distance between the correct and the given answer. Thus, the bigger the error-angle was, the greater the video rotation. We performed training to eliminate learning and habituation effects. Participants were trained using 10 trials of each spatial task.

#### Experimental phase inside the MR imager

***Procedure***. The participants were told that they had to perform the same tasks as before but in the scanner. The trials were displayed using E-prime software (E-prime Psychology Software Tools Inc., Pittsburgh, PA, USA) synchronized via the signals from the scanner. A back-projection screen, received the images, and a mirror, attached to the head coil, allowed the participant to view the screen. After being positioned within the magnet, participants were shown an example of each trial performed during the training phase without concurrent fMRI recording to familiarize them with performing the task while in a horizontal position. Participants indicated their response on an MR-compatible joystick that was positioned on the upper part of the right leg. Participants moved the joystick with the right hand and pushed a button with the left hand when the joystick was oriented in the desired direction. Angle errors were recorded during the test phase using VRML-prime. Participants were not given any feedback about their performance. All participants were presented with the spatial tasks in the same block order as in the training phase (e.g., A, ERO, EU, and C, counterbalanced across participants). When grouping the encoding and test phase, each trial lasted 27 s with an inter-trial interval of 3 s. Participants underwent two functional scans (of four blocks each) with five trials of each conditions. The duration of the functional scan was thus 9 min, following a brief period during which the scanner reached equilibrium. In this phase, five trials of each condition were consecutively presented in a random order and formed a block. The order of the blocks was repeated twice for each participant. The block order was counterbalanced across participants. For each functional scan, 200 functional volumes were acquired.

***MR acquisition***. The experiment was performed in a whole-body 3T MR scanner (Bruker MedSpec S300) with 40 mT/m gradient strength. For functional scans, the manufacturer-provided gradient-echo/T2^*^ weighted EPI method was used. Thirty-nine adjacent axial slices parallel to the bi-commissural plane were acquired in the interleaved mode. Slice thickness was 3.5 mm. The in-plane voxel size was 3 × 3 mm (216 × 216 mm field of view acquired with a 72 × 72 pixels data matrix; reconstructed with zero filling to 128 × 128 pixels). The main sequence parameters were: *TR* = 3 s, *TE* = 30 ms, flip angle = 77°. To correct images for geometric distortions induced by local B0-inhomogeneity, a B0 fieldmap was obtained from two gradient echo data sets acquired with a standard 3D FLASH sequence (Δ*TE* = 9.1 ms). The fieldmap was subsequently used during data processing. Finally, a T1-weighted high-resolution three-dimensional anatomical volume was acquired, by using a 3D Modified Driven Equilibrium Fourier Transform (MDEFT) sequence (field of view = 256 × 224 × 176 mm; resolution: 1.333 × 1.750 × 1.375 mm; acquisition matrix: 192 × 128 × 128 pixels; reconstruction matrix: 256 × 128 × 128 pixels).

***Data processing***. Data analysis was performed by using the general linear model as implemented in SPM5 (Welcome Department of Imaging Neuroscience, London, UK, www.fil.ion.ucl.ac.uk/spm) where each event was modeled using a hemodynamic function model. Data analysis started with the spatial pre-processing steps. First, the functional volumes were time-corrected (slice timing) with the 19th slice as the reference, in order to correct for effects induced by the different acquisition time of each slice within the functional volume. Subsequently, all volumes were realigned to correct for head motion using rigid body transformations. After discarding the first four slices, while the scanner reached equilibrium, the first volume of the first ER-fMRI session was taken as the reference volume (i.e., this volume was originally the fifth volume). Unwarping was performed by using the individually acquired fieldmaps, to correct for interaction between head movements and EPI distortions. T1-weighted anatomical volume was co-registered to mean images created by the realignment procedure and was normalized to the MNI space using a trilinear interpolation. The anatomical normalization parameters were subsequently used for the normalization of functional volumes. Finally, each functional volume was smoothed by an 8-mm FWHM (Full Width at Half Maximum) Gaussian kernel to improve differences in intersubject localization. Time series for each voxel were high-pass filtered (1/128 Hz cutoff) to remove low frequency noise and signal drift.

***Statistical analysis of neuroimaging data***. After spatial pre-processing steps, the statistical analysis was first performed separately on the functional images acquired for each task during the first 18 s of each trial (reflecting the encoding phase of a trial). The cerebral activity during the test phase of each trial was not included in the present study, thus, the functional images from the test phase were discarded from the analysis. The conditions of interest (A, EU, ERO, and C) were modeled as 4 regressors convolved with a canonical hemodynamic response function (HRF). The movement parameters derived from the realignment corrections (3 translations and 3 rotations) were also entered in the design matrix as additional factors. The trial performance (i.e., error angle) was entered as a parametric modulator of each trial. The general linear model was then used to generate the parameter estimates of the activity for each voxel, each condition and each participant. Statistical parametric maps were generated from linear contrasts between the HRF parameter estimates for the different experimental conditions. The spatial resolution of the statistical parametric maps was the same as the spatial resolution of the functional MR acquisition (3 × 3 × 3.5 mm). Several statistical analysis have been performed as follows: Approximate AR(1) autocorrelation model estimated at omnibus *F*-significant voxels (*p* < 0.001), was used over the whole brain. Specific effects were tested with the appropriate linear contrasts of the parameter estimations, and the corresponding contrast images were subsequently entered into a random effects analysis.

Group analysis was performed with the contrast images provided by the individual analyses (Friston et al., 1998). The random effects analysis at the group level can be divided as follows depending on the theoretical question underlying the analysis:

- Global spatial encoding network
  First, the main contrasts assessed the correlates of spatial encoding for each spatial condition A, EU, ERO vs. the C condition. Then, the conjunction analysis assessed the neural correlates common to all spatial encoding tasks. For this purpose, we applied an inclusive masking for all three main contrasts (Friston et al., 2005).
- Differenciating neural correlates of spatial encoding conditions
  First, the main contrasts assessed the questions of interest in this study. EU vs. A provides cerebral substrate engaged by egocentric referencing and due to self-motion information (i.e., idiothetic). A vs. EU provides information about landmark-based information (i.e., allothetic). ERO vs. EU provides information related to the cerebral substrate of rotation processing while EU vs. ERO reveals cerebral network of the egocentric-updating due to self-motion information. We also calculated the contrasts: ERO vs. A and A vs. ERO.

Subsequently, a multiple regression analysis was performed. Specifically, we included individual contrast images (one image per participant) reflecting the mean activation for each previously mentioned contrasts.

Moreover, we also performed a correlation analysis to assess the task-specific regions modulated by the performance. For this purpose, we calculated correlations between the BOLD signal in task-specific regions and the performance of task-execution. This analysis is supposed to reflect the regions which were closely related to spatial processing (Maguire et al., 1998; Wolbers and Buchel, 2005; Wolbers et al., 2007, 2008; Baumann et al., 2010). The evaluated performance was the average angle error size (i.e., difference between the expected pointing direction and the observed pointing direction). The average error size for each participant in each condition was the variable included in a multiple regression analysis. As the correct performance is reflected by the small size of the angle error, the regions showing anti-correlation between BOLD and performance, were considered specifically related to the achievement of spatial processing.

For all our analyses, we used an extent threshold of 10 contiguous voxels, and a voxel-level height threshold of *p* < 0.001, uncorrected for contrasts (height threshold: *T* = 3.55). The extended threshold of 10 voxels was determined empirically and then used for all contrasts. However, as advised by Bennett et al. (2009) FDR threshold are provided in the Tables to provide corrected values detailing the prevalence of false positives. An in-house modification of the “spm_list.m” file, including a non-linear transform of MNI to Talairach (http://imaging.mrc-cbu.cam.ac.uk/imaging/MniTalairach), allowed us to visualize both MNI and Talairach coordinate in the SPM result. From the displayed Talairach coordinate, anatomical locations were determined using Talairach Daemon software (http://www.talairach.org/, see also Lancaster et al., 2000), and checked with the Talairach and Tournoux paper atlas (Talairach and Tournoux, 1988).

Analyses were also performed with small volume correction (SVC) for multiple comparisons (*P* < 0.05, corrected) in ROIs. In this study, we were particularly interested in the role of the hippocampus and retrosplenial areas. Therefore, predefined anatomic labels (WFU PickAtlas Tool, http://www.fmri.wfubmc.edu/download.htm) were applied to identify each of the following ROI: hippocampus (left and right) and Brodmann area 30, 31 (left and right).

## Results

### Behavioral results

Table 1 summarizes the performance values obtained for each condition. An ANOVA conducted on the angular error with conditions as a within-subject variable (A, EU, ERO, C) showed a main effect for experimental conditions [*F*_(3, 51)_ = 35.2, *MSE* = 121.64, *partial* η^2^ = 0.67, *p* < 0.001]. Specific contrast of interest A vs. EU, EU vs. ERO, and ERO vs. A were not statistically significant [*F*_(1, 17)_ = 1.76, *MSE* = 98.63. *p* = 0.20, and *F*_(1, 17)_ = 3.60, *MSE* = 186.9. *p* = 0.07, and *F*_(1, 17)_ = 1.15, *MSE* = 143.06. *p* = 0.30, respectively]. *Post-hoc* Bonferroni comparisons only showed that smaller pointing errors were made in the C condition (mean = 3.4°, *SD* = 3.5°) compared to all other spatial conditions (mean = 33.4°, *SD* = 13.5°, *p*s < 0.001, all other *p*s are non-significant).

**Table 1:** **Average angle size error (with standard deviations in parentheses) in the object pointing task for each condition**.

**BEHAVIORAL PERFORMANCE**	
**Condition**	**Average error angle size (standard deviation)**
Allocentric	33.34 (12.01)
Egocentric	
Updating	37.73 (15.48)
Rotation only	29.08 (11.95)
Control	3.36 (3.37)

*The A condition (with an object-to-object task), the EU and the ERO conditions (with a self-to-object task), and the C condition (with a straightforward pointing task)*.

A correlation matrix of spatial performance for each spatial condition revealed a significant correlation between performance values in A and EU conditions (*r* = 0.5, *p* < 0.05). Participants who performed well in the A condition were also good performers in the EU condition. However, the ERO performance was not significantly correlated with A or with EU performance which suggests that those who performed well in the A or EU condition did not necessarily perform well in the ERO condition.

### fMRI results

#### Global spatial encoding network

***Allocentric (A) encoding***. As illustrated in the Supplementary Material file—Table S1, the contrast A vs. C revealed a large cluster of 4128 activated voxels with an activation peak located within the left cuneus (BA 17). The activation included the bilateral temporal lobe, the bilateral hippocampus, the bilateral parietal lobe (superior parietal lobule, BA 7 and inferior parietal lobule, BA 40), frontal areas (BA 4), and the cerebellum.

***Egocentric-updating (EU) encoding***. As illustrated in the Supplementary Material file—Table S2, the contrast EU vs. C revealed a large cluster of 4086 activated voxels with an activation peak within the left hippocampus. Other activations were observed in the bilateral parietal lobe (superior parietal lobule, BA 7 and inferior parietal lobule, BA 40), cingulate gyrus (BA 31), the right frontal area (BA 4), and the cerebellum.

***Egocentric with Rotation Only (ERO) encoding***. As illustrated in the Supplementary Material file—Table S3, the contrast ERO vs. C revealed a large cluster of 5381 activated voxels with an activation peak located within the left cuneus (BA 17), extending within the bilateral temporal lobe. Other activations were observed in the bilateral parietal lobe (precentral gyrus, BA 4; gyrus-post-central, BA 2, 3), the frontal areas (BA 4, 6), and the cerebellum.

***Conjunction analysis***. The conjunction analysis identified the common activation (Table 2) of spatial encoding. They were located in the right cuneus, BA 18, right hippocampus and left superior parietal lobule, BA 7.

**Table 2:** **Activated regions for spatial encoding commonly activated for A, EU, ERO, vs. C (Conjunction analysis, statistical threshold: uncorrected *p* < 0.001, cluster extent: *k* ≥ 10 voxels)**.

**Cerebral activated regions**	**Side**	**BA**	***k***	**Talairach coordinates (*x, y, z*)**			***T*-value**	**FDR corrected threshold**
**OCCIPITAL CORTEX**								
Cuneus	R	BA 18	3062	18	−96	11	6.31	0.000
**TEMPORAL CORTEX**								
Hippocampus	R	–	125	18	−32	5	5.86	0.001
**PARIETAL CORTEX**								
Superior parietal lobule	L	BA 7	108	−27	−55	58	4.56	0.001

*The Talairach coordinates (x, y, z) are indicated for each voxel. The side, Right (R) and Left (L), gyri and Brodmann areas (BA) are mentioned. Abbreviation: A, Allocentric; EU, Egocentric-updating; ERO, Egocentric with Rotation Only; C, Control. FDR corrected threshold provides corrected values detailing the prevalence of false positives in the analysis*.

#### Differenciating neural correlates of spatial encoding conditions

The following contrasts revealed activation for the spatial condition, without considering the retrieval performance of subjects.

**Figure 2:**
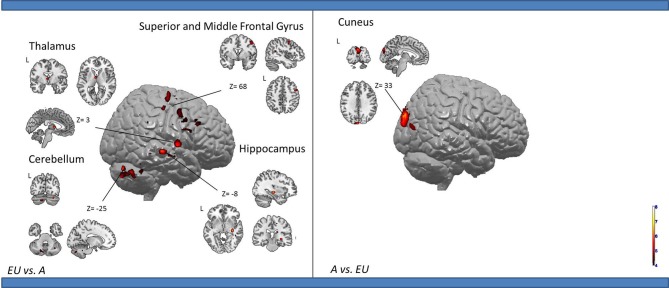
**Shows regions reported in Table 3 projected onto 3D anatomical template (random-effect analysis, statistical threshold: uncorrected *p* < 0.001, cluster extent: *k* ≥ 10 voxels)**. Activations are provided by a random effect analysis, for (1) EU spatial encoding when contrasted with the A condition (on the left), and (2) A spatial encoding when contrasted with EU (on the right). Clusters are projected on 2D anatomical slices (T1-template image) in neurological convention. L is left hemisphere. The color scale represents the *t*-value of activation.

***Egocentric-updating compared to Allocentric condition (Table 3, Figure 2)***. *EU vs. A* revealed activation in bilateral frontal regions (such as the superior and middle frontal gyri, BA 6, 8, 9, 32, 46), left thalamus, and bilateral cerebellum. More importantly, we observed that the right hippocampus was specifically activated for *EU vs. A*.

**Table 3:**
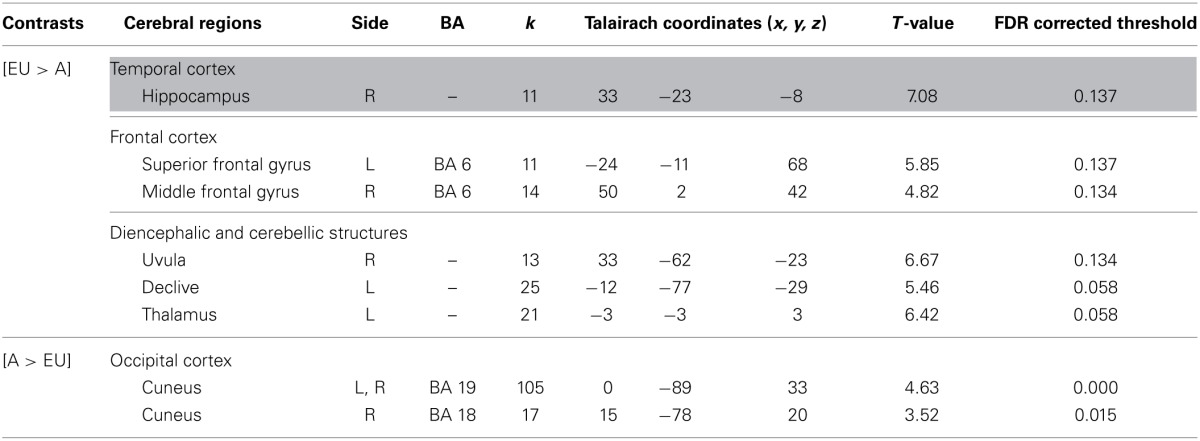
**Activated regions due to self-related changes in location and orientation during spatial encoding, provided by the contrast: (1) EU vs. A for the upper part of the table; (2) A vs. EU for the lower part of the table (random-effect analysis, uncorrected *p* < 0.001, *k* ≥ 10 voxels)**.

*The right hippocampal activation in the EU condition is highlighted. The Talairach coordinates (x, y, z) are indicated for each voxel. The side, Right (R) and Left (L), gyri and Brodmann areas (BA) are mentioned. Regions reported in both contrasts are highlighted. FDR corrected threshold provides corrected values detailing the prevalence of false positives in the analysis*.

*A vs. EU* revealed an activation of the bilateral cuneus (BA 18, 19).

We then tested our a-priori hypotheses about a differential hippocampal activation pattern for both conditions (FDR small-volume corrected, *p* < 0.05) and observed that the right hippocampus was activated in the EU condition (Table 6).

**Figure 3:**
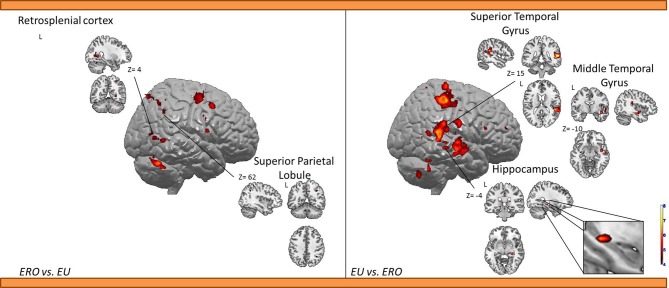
**Shows regions reported in Table 4 projected onto a 3D anatomical template (random-effect analysis, statistical threshold: uncorrected *p* < 0.001, cluster extent: *k* ≥ 10 voxels)**. On the left, ERO is contrasted to EU; on the right, EU is contrasted to ERO. The retrosplenial activity, along with the superior parietal gyrus activations are highlighted for the right contrast. The right hippocampal activity (cluster extent *k* = 8 voxels), along with the superior and inferior temporal gyrus activations are highlighted on the right contrast. These clusters are projected onto 2D anatomical slices (T1-template image) in neurological convention. L is left hemisphere. The color scale represents the *t*-value of activation.

***Egocentric with Rotation only compared to Egocentric-updating condition (Table 4, Figure 3)***. *ERO vs. EU* activated the right retrosplenial cortex (BA 30), superior parietal lobule (BA 7). Supplementary activity was observed in the bilateral middle and medial frontal gyrus (BA6, 32), right thalamus, and bilateral cerebellum.

**Table 4:**
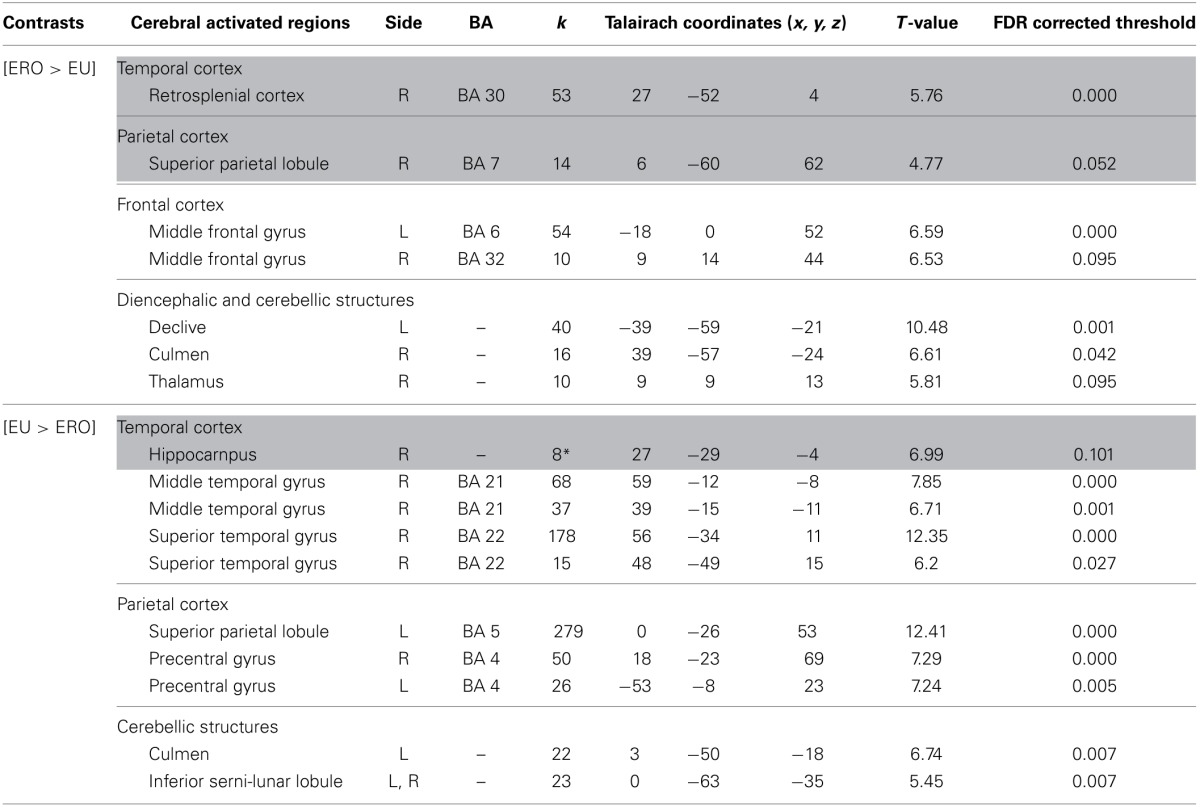
**Activated regions for differential effects within two types of Egocentric spatial encoding, rotation changes vs. orientation and location changes**.

They were provided by the contrast: (1) ERO vs. EU for the upper part of the table. (2) EU vs. ERO for the lower part of the table (random-effect analysis, uncorrected p < 0.001, k ≥ 10 voxels, ^*^ for the hippocampus k = 8). The Talairach coordinates (x, y, z) are indicated for each voxel. The side, Right (R) and Left (L), gyri and Brodmann areas (BA) are mentioned. FDR corrected threshold provides corrected values detailing the prevalence of false positives in the analysis.

*EU vs. ERO* revealed an activation of the right hippocampus (8 voxels), left superior parietal lobule (BA 5), and right middle and superior temporal gyrus (BA 21, 22). Supplementary activity was observed in the bilateral precentral gyrus (BA 4) and cerebellum.

We then tested our a-priori hypotheses about a differential retrosplenial and hippocampal activation pattern for both conditions (FDR small-volume corrected, *p* < 0.05). We observed that the right Brodmann area 30 was activated in the ERO condition while the right hippocampus was activated in the EU condition (Table 6).

**Figure 4:**
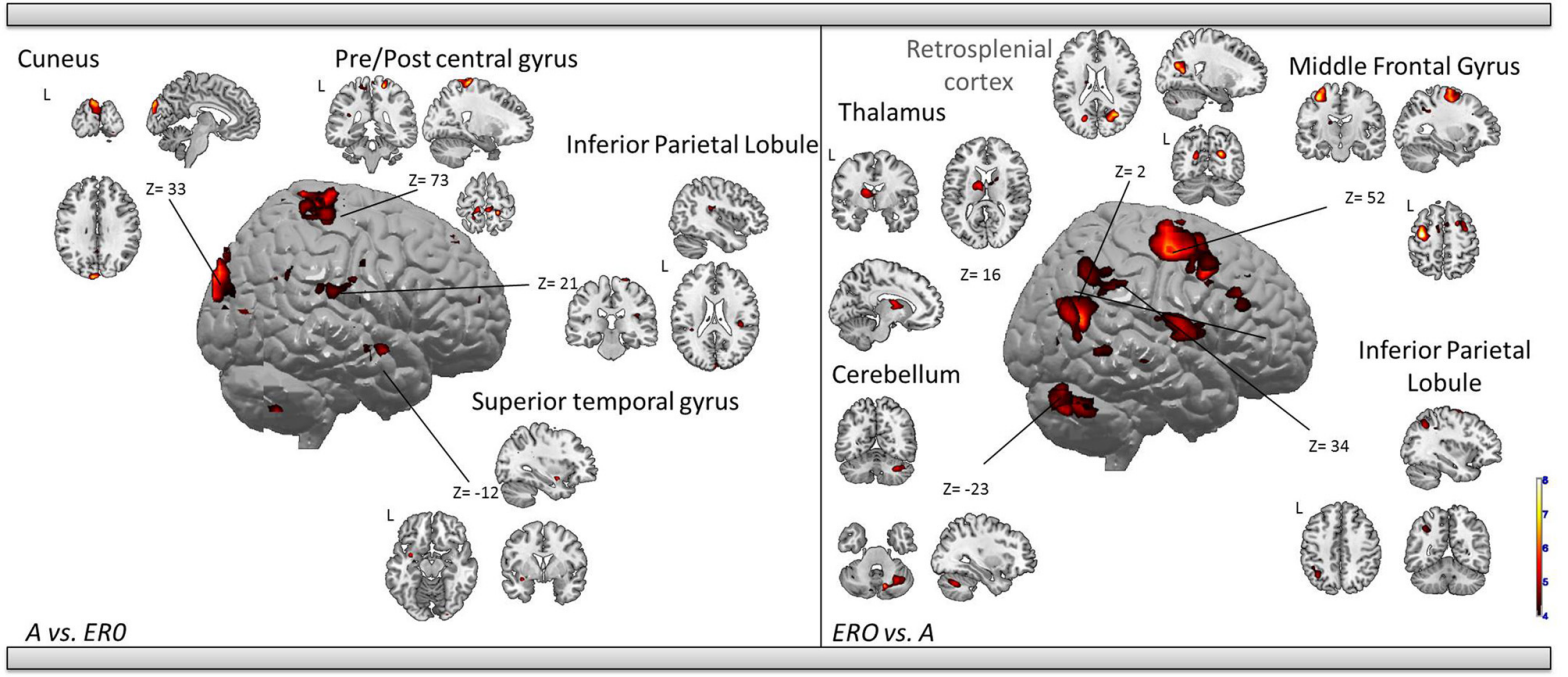
**Shows regions reported in Table 3 projected onto a 3D anatomical template (random-effect analysis, statistical threshold: uncorrected *p* < 0.001, cluster extent: *k* ≥ 10 voxels)**. Activations are provided by a random effect analysis, for the A spatial encoding when contrasted (1) with the EU condition (on the left), and (2) with the ERO condition (on the right). Occipital activity in the cuneus which is observed in both contrasts is highlighted. These clusters are projected onto 2D anatomical slices (T1-template image) in neurological convention. L is left hemisphere. The color scale represents the *t*-value of activation.

***Allocentric compared to Egocentric with Rotation only condition (Table 5, Figure 4)***. *A vs. ERO* also revealed activation of the bilateral cuneus (BA 18, 19). Supplementary activations were observed in the left superior temporal gyrus (BA 34, 38), right superior (BA 5) and inferior (BA 40) parietal lobule, bilateral frontal (pre-central gyrus, BA 4), and right cerebellum.

**Table 5:**
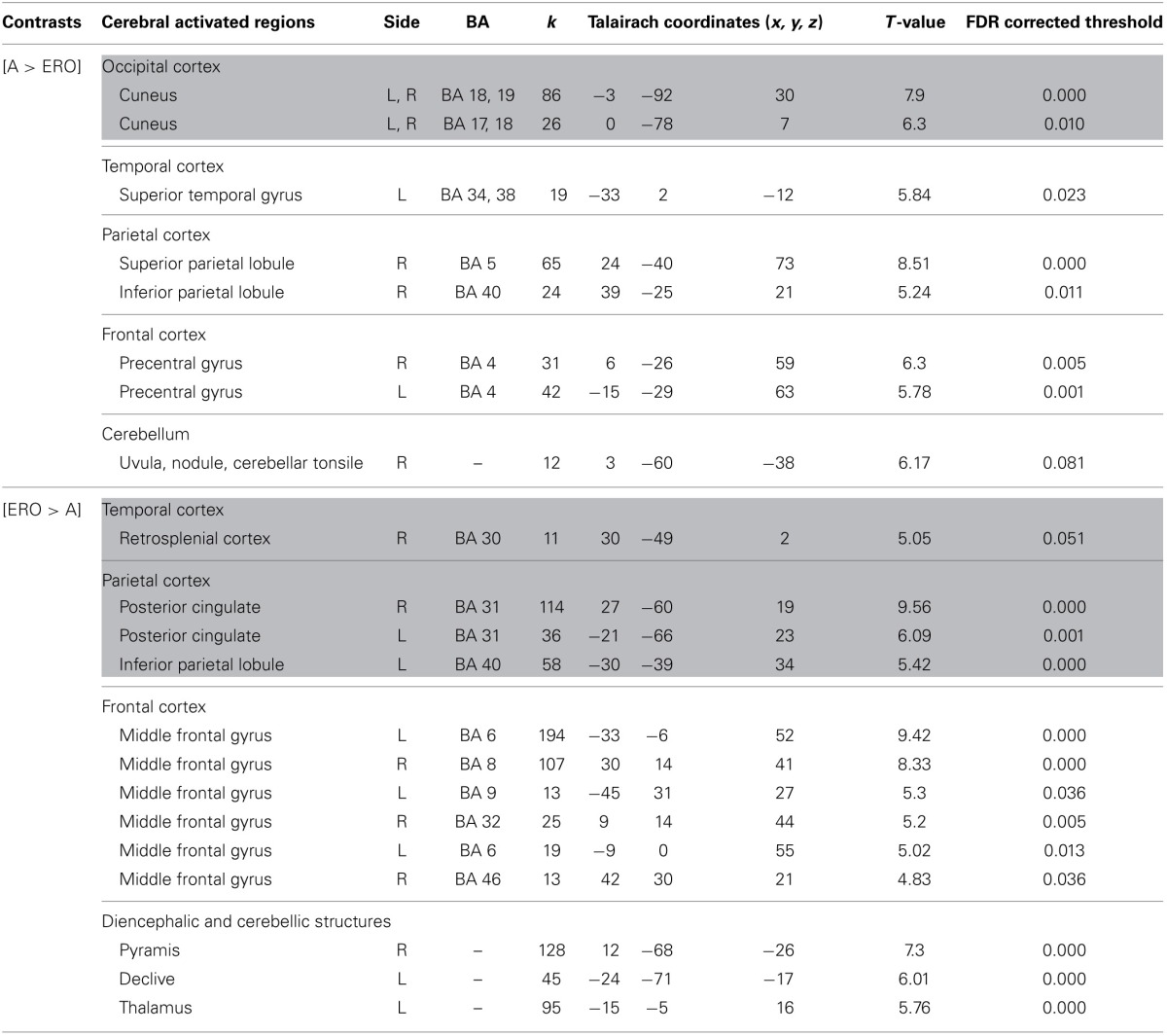
**Activated regions provided by the contrast: (1) A vs. ERO for the upper part of the table; (2) ERO vs. A for the lower part of the table (random-effect analysis, uncorrected *p* < 0.001, *k* ≥ 10 voxels)**.

*The Talairach coordinates (x, y, z) are indicated for each voxel. The Hemisphere, Right (R) and Left (L), gyri and Brodmann areas (BA) are mentioned. Regions reported in both contrasts are highlighted. FDR corrected threshold provides corrected values detailing the prevalence of false positives in the analysis*.

*ERO vs. A* revealed specific activation in the right retrosplenial cortex (BA 30) extended to the bilateral posterior cingulate (BA 31) and left inferior parietal lobule (BA 40). Supplementary activities were observed in the bilateral frontal regions (superior and middle frontal gyri, BA 6, 8, 9, 32, 46), left thalamus, and bilateral cerebellum.

We then tested our a-priori hypotheses about a differential retrosplenial activation pattern for both conditions (FDR small-volume corrected, *p* < 0.05). We observed that the right Brodmann area 30 and Brodmann area 31 were activated in the ERO condition (Table 6).

**Table 6:** **Hippocampal and retrosplenial activation in the FDR small-volume corrected analysis (*p* < 0.05) of spatial encoding conditions**.

**Contrasts**	**Cerebral regions**	**Side**	***BA***	**Talairach coordinates (*x, y, z*)**			***T*-value**
[EU > A]	Hippocampus	R	–	30	−23	−5	5.21
[ERO > EU]	Retrosplenial cortex	R	BA 30	24	−52	0	5.69
[EU > ERO]	Hippocampus	R	–	24	−29	−4	6.99
[ERO > A]	Retrosplenial cortex	R	BA 30	15	−59	9	4.72
	Retrosplenial cortex	R	BA 31	12	−68	21	5.68

*The Talairach coordinates (x, y, z) are indicated for each voxel. The Hemisphere, Right (R) and Left (L), gyri and Brodmann areas (BA) are mentioned*.

***Correlation analysis***. Among the A-specific regions, no regions were anticorrelated with the pointing errors. Among the EU-specific regions, only the junction between the right hippocampus and caudate nucleus (peak coordinates: 21, −40, 8, *T* = 5.47, see Figure S1 from Supplementary Material) was significantly anticorrelated with the pointing errors (*r* = −0.87). A small-volume correction analysis (FWE corrected, *p* < 0.05) on the right hippocampus confirmed that the right hippocampus was significantly correlated (peak coordinates: 16, −33, 11). Finally, among the ERO-specific regions, no regions were anticorrelated with the pointing errors.

## Discussion

Our results confirmed the activation of a large occipito-parieto-temporal network common for spatial encoding conditions (e.g., Ghaem et al., 1997). Beyond the classical allocentric-egocentric distinction, the type of referencing induced by the visual perspective during encoding and the task-demand, allowed the identification of essential regions within the spatial memory network. The major result was the specific involvement of two areas during the self-related conditions, which were the retrosplenial cortex for orientation changes, and the right hippocampus for self-location changes.

The first crucial result of the study reveals an enhanced retrosplenial activity when contrasting ERO to EU. Encoding new spatial information during the ERO condition only relied on rotation changes, while, during the EU condition, the weight of rotation information during the integration of new spatial information was weakened by the concomitant change of location. This result therefore provides evidence that the retrosplenial cortex in humans specifically processes changes in head direction. Moreover, although the performances for A and EU conditions were correlated, the ERO performance was not correlated with the allocentric or the egocentric-updated performance. Therefore, participants that were good at processing head direction changes were not necessarily good at processing location changes or allocentric landmark-based information. This supports the idea that relatively independent cognitive mechanisms are at play in those tasks.

Although the retrosplenial cortex (BA 30) is known to contain head-direction cells in rodents (Cho and Sharp, 2001; Sharp et al., 2001; Wiener and Taube, 2005), only limited experimental evidence was reported in humans. Concerning clinical evidence, it has been suggested that right retrosplenial lesions lead to pure topographical disorientations (Valenstein et al., 1987; Yasuda et al., 1997; Aguirre and D'Esposito, 1999; Maguire, 2001; Vann and Aggleton, 2004; Vann et al., 2009), reflecting a type of heading disorientation (i.e., an inability to represent the orientation direction with respect to the external environment).

Functional MRI studies have provided evidence suggesting that the retrosplenial cortex was strongly activated during scene viewing, scene imagery, and scene memory (Epstein et al., 2007). Moreover, a meta-analysis of navigation fMRI studies reported bilateral involvement of BA 30 in humans (Maguire, 2001; for an extensive review see Vann et al., 2009). Congruently, we report a common spatial encoding network encompassing the bilateral retrosplenial cortex (when compared to control condition). Overall, these results make it possible to pinpoint the implication of the retrosplenial region in spatial processing.

However, with regards to head-direction, only one fMRI study aimed at identifying neural correlates of orientation This study reported a region situated close to the anatomically defined retrosplenial cortex (BA 30, see Maguire, 2001, for a review), in BA 31 (Baumann and Mattingley, 2010). In our current study we report for the first time the activation of the right retrosplenial cortex (BA 30) for the rotation. This result is in agreement with animal studies of spatial encoding. Additionally, we observed the BA 30 activation during ERO vs. A contrast, next to the bilateral posterior cingulate (BA 31), in line with Baumann and Mattingley (2010)'s findings.

An influential model of spatial memory proposes that the retrosplenial cortex supports, in humans, stimulus conversion from an egocentric reference frame (in the parietal cortex) to an allocentric reference frame (in the medial temporal regions) and vice versa (Burgess et al., 2001a,b; Burgess, 2008; Vann et al., 2009). However, with regards to retrosplenial function, this model was mainly based on animal studies evidence. Few fMRI studies have reported retrosplenial activity congruent with this model (Lambrey et al., 2012; Zhang et al., 2012). Our result supports the hypothesis of retrosplenial cortex encompassing cells activated for head-direction in humans. Neuronal electrical recordings in humans are necessary to provide further direct support of the retrosplenial involvement.

The second crucial activated region reported in this study is the right hippocampal area. It was preferentially involved in spatial encoding, which required an online integration of self-location transformation in the spatial representation, rather than encoding the schematic spatial relation amongst objects (O'Keefe and Nadel, 1978). In fact, the contrast of the EU condition to the A condition significantly enhanced the right hippocampal activity. This result is in line with previous observations of right hippocampal activation during path integration encoding in humans (Wolbers et al., 2007). Nonetheless, during retrieval, the left hippocampal structure might be further involved, as suggested by Iglói et al. (2010). Moreover, this hippocampal activation is congruent with Shelton and Gabrieli (2002)'s results which compared cerebral activities during encoding from a route perspective and a survey perspective. They observed bilateral hippocampal activation specific to the route condition. Differences in the type of subsequent task-demand (recognition task vs. spatial self-to-object pointing in this study) and the environment complexity (large with multiple boundaries and 17 landmarks vs. medium with an average of 5 landmarks in this study) can account for the right lateralization of the hippocampal activation in the present study.

Moreover, the modulation by between-subject retrieval performance of a hippocampal-caudate cluster observed in the correlation analysis indicates a critical role for these structures in the subsequent task-resolution. During encoding, good egocentric-updating pointers showed stronger hippocampal activation (extending to caudate). The hippocampal activity has previously been reported to correlate to accuracy of self-location or self-orientation, in a path integration task (Wolbers et al., 2007, using an egocentric pointing task) or during active navigation, (Maguire et al., 1998; Hartley et al., 2003). We therefore interpret this hippocampal activity in good memory performers as highlighting the importance of the right hippocampus for processing self-location changes.

Because the EU vs. A contrast provides the neural substrate engaged by the egocentric-updating and due to self-motion information (i.e., idiothetic) rather than landmark-based information, we suggest that the right hippocampal region may be more specifically devoted to location related self-related changes. Additionally, because allocentric processing such as landmarks and room geometry processing were likely to occur in the EU condition as well, it is possible that the hippocampal involvement could also arise from the relative flexibility of the representation constituted during the EU encoding compared to the more schematic representation during the A encoding. In fact, Zhang and Ekstrom (2013) have shown that such differences in the use of an allocentric representation could be responsible for a differential hippocampal involvement in favor of a flexible representation. As this right hippocampal activity is also observed when directly contrasting EU vs. ERO within egocentric referencing, it is likely that self-motion location change in the EU condition plays a crucial role in this hippocampal activation. This additional evidence supports the hypothesis that, in humans, location self-related changes involve the right hippocampal region.

This result is new and central, as a wide body of evidence from animal literature, and in particular the discovery of place cells, have led to the proposal that the hippocampal structure underlies cognitive mapping in an allocentric reference frame (O'Keefe and Dostrovsky, 1971; O'Keefe and Nadel, 1978; O'Keefe et al., 1998). In humans, single cells neuron recordings have shown that place cells exist in the human hippocampus (Ekstrom et al., 2003).

In the present experiment, we report substantial hippocampal activation when participants must encode allocentric landmark-based spatial information (there is a significant activation of the hippocampus in the A encoding condition compared to the C encoding condition). However, given previous neuropsychological results, we expected that the hippocampal structure could be enhanced when idiothetic self-location changes are involved (Worsley et al., 2001; Philbeck et al., 2004; Gomez et al., 2012). From a behavioral perspective, participants who performed well in the EU condition also performed well in the A condition. Together with the conjunction results, this observation supports the hypothesis that a unique structure, the hippocampus, may underlie different spatial processing, in the present case, egocentric-updating and allocentric processing, which could explain this correlational effect.

Overall, these findings support models emphasizing the need to combine allocentric landmark sensory aspects to egocentric-updating to form the place code of Place cells (e.g., Redish and Touretzky, 1997). Several proposals have suggested that the hippocampus might be involved in complementary types of spatial processing such as path integration processing (Redish and Touretzky, 1997; Whishaw et al., 1997, 2001; Worsley et al., 2001; Wolbers et al., 2007). These models have underlined that the hippocampal structure is well-suited to underlie such spatial processing as it is interconnected to various structures making it possible to represent, together, the body's position: it receives input from systems assumed to represent head-direction (such as the retrosplenial cortex, and thalamus), and from systems processing self-motion, such as the human motion complex (Redish and Touretzky, 1997; Wiener and Taube, 2005).

In closing, our results refine previous findings on navigation in large-scale environments (Maguire et al., 1998; Ekstrom et al., 2003; Hartley et al., 2003) by suggesting that hippocampal activity should be extended to represent self-related location transformations. In keeping with existing models (Burgess et al., 2001a; Buzsaki, 2005), we suggest that, in humans, during spatial encoding: (1) the retrosplenial cortex processes heading-direction; (2) the hippocampus processes self-related location transformations and combines it with landmark information to allow place computations. Such a combination would allow for correcting accumulated errors during egocentric-updating.

To conclude, this experiment highlighted that, in some circumstances determined by the experimental conditions, hippocampal and retrosplenial structures known to be involved in allocentric environmental coding (Galati et al., 2010) could demonstrate a preferential involvement in an egocentric coding of space. Consequently the differentiation between allocentric vs. egocentric representation no longer seems to be sufficient in understanding the complexity of the mechanisms at play during spatial encoding.

### Conflict of interest statement

The authors declare that the research was conducted in the absence of any commercial or financial relationships that could be construed as a potential conflict of interest.
