# Supplementary material for: Differential hippocampal and retrosplenial involvement in egocentric-updating, rotation, and allocentric processing during online spatial encoding: an fMRI study
Source: Front Hum Neurosci. 2014 Mar 20;8:150. doi: 10.3389/fnhum.2014.00150 (PMC3960510; doi:10.3389/fnhum.2014.00150)
Supplement: Supplementary file 1 [file DataSheet1.ZIP › SupplementaryMaterial-Gomez/75989_Gomez_Table_7.PDF]

Contrasts	Cerebral activated regions	Side	BA	k	Talairach coordinates (x, y, z)			T value	FDR corrected threshold
[A> C]	*Occipital cortex*								
	Cuneus	L	BA 17	4128	-3	-93	2	20.98	0.000
	Temporal cortex								
	Hippocampus	R	-	117	18	-35	5	13.3	0.000
	Hippocampus	L	-	26	-36	-9	-11	7.14	0.009
	Parietal cortex								
	Superior Parietal Lobe	L	BA 7	88	-24	-58	67	7.38	0.000
	Inferior Parietal Lobule	L	BA 40	22	-42	-28	27	6.61	0.004
	Inferior Parietal Lobule	R	BA 40	47	36	-28	24	6.46	0.000
	Frontal cortex								
	Precentral Gyrus	L	BA 4	30	-53	-16	36	7.68	0.006
	Paracentral Lobule	L	BA 4	339	-18	-37	66	6.92	0.000
	Paracentral Lobule	L	BA 4	17	-21	-27	46	6.46	0.027
	Paracentral Lobule	R	BA 4	25	12	-24	50	5.67	0.009
	Cerebellic structures								
	Uvula	L, R	-	22	0	-63	-32	6.59	0.012
	Cerebellar Nodule	L	-	15	-6	-42	-30	4.97	0.035
