# Supplementary material for: Differential hippocampal and retrosplenial involvement in egocentric-updating, rotation, and allocentric processing during online spatial encoding: an fMRI study
Source: Front Hum Neurosci. 2014 Mar 20;8:150. doi: 10.3389/fnhum.2014.00150 (PMC3960510; doi:10.3389/fnhum.2014.00150)
Supplement: Supplementary file 1 [file DataSheet1.ZIP › SupplementaryMaterial-Gomez/75989_Gomez_Table_8.PDF]

Contrasts	Cerebral activated regions	Side	BA	k	Talairach coordinates (x, y, z)			T value	FDR corrected threshold
[EU > C]									
	Temporal cortex								
	Hippocampus	L	-	4086	-21	-35	5	12.43	0.000
	Parietal cortex								
	Superior Parietal Lobule	L	BA 7	83	-33	-58	58	9.59	0.000
	Superior Parietal Lobule	L	BA 7	37	-15	-40	73	8.89	0.003
	Inferior Parietal Lobule	R	BA 40	48	36	-28	24	8.91	0.001
	Cingulate Gyrus	L	BA 31	23	-21	-24	43	9.16	0.016
	Frontal cortex								
	Paracentral Lobule	R	BA 4	29	9	-31	69	6.15	0.007
	Paracentral Lobule	R	BA 4	22	15	-21	46	5.18	0.017
	Cerebellic structures								
	Uvula, Nodule, Cereb. Tonsile	L, R	-	35	0	-63	-32	6.62	0.003
