# Supplementary material for: Differential hippocampal and retrosplenial involvement in egocentric-updating, rotation, and allocentric processing during online spatial encoding: an fMRI study
Source: Front Hum Neurosci. 2014 Mar 20;8:150. doi: 10.3389/fnhum.2014.00150 (PMC3960510; doi:10.3389/fnhum.2014.00150)
Supplement: Supplementary file 1 [file DataSheet1.ZIP › SupplementaryMaterial-Gomez/75989_Gomez_Table_9.PDF]

Contrasts	Cerebral activated regions	Side	BA	k	Talairach coordinates (x, y, z)			T value	FDR corrected threshold
[ERO> C]									
	Occipital cortex								
	Cuneus	L	BA 17	5381	-9	-93	-1	17.32	0.000
	Parietal cortex								
	Precentral Gyrus	L	BA 4	12	-33	-9	55	4.83	0.121
	Postcentral Gyrus	R	BA 2, 3	31	36	-33	47	5.99	0.007
	Frontal cortex								
	Paracentral Lobule	L	BA 4	19	-9	-31	73	6.31	0.038
	Superior Frontal Gyrus	R	BA 6	48	24	0	64	6.27	0.001
	Cerebellic structures								
	Uvula, Nodule, Cereb. Tonsile	L, R	-	11	0	-63	-32	5.5	0.125
