# Supplementary figures and images for: Differential hippocampal and retrosplenial involvement in egocentric-updating, rotation, and allocentric processing during online spatial encoding: an fMRI study
Source: Front Hum Neurosci. 2014 Mar 20;8:150. doi: 10.3389/fnhum.2014.00150 (PMC3960510; doi:10.3389/fnhum.2014.00150)

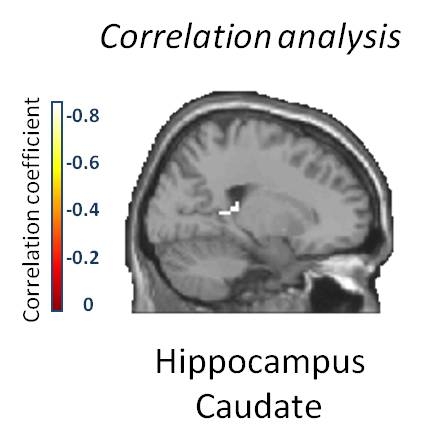

Supplement: Supplementary file 1 [file DataSheet1.ZIP › SupplementaryMaterial-Gomez/75989_Gomez_Figure_5.JPEG]
